# Assessment of oxidative stress biomarkers in *Palaemon varians* exposed to deep eutectic systems

**DOI:** 10.1007/s11356-024-34983-3

**Published:** 2024-09-21

**Authors:** Mª Pilar Garralaga, Ines Ferreira, Laura Lomba, Elisabet Pires, Sara Gracia-Barberán, Ana Rita C. Duarte, Mário Diniz

**Affiliations:** 1 Universidad San Jorge. Campus Universitario, Autov A23 Km 299, 50830 Zaragoza, Villanueva de Gállego Spain; 2 LAQV-REQUIMTE, Department of Chemistry, School of Science and Technology, NOVA University Lisbon, 2829-516 Caparica, Portugal; 3 Instituto de Síntesis Química y Catálisis Homogénea (ISQCH), Facultad de Ciencias, CSIC-Universidad de Zaragoza, C/ Pedro Cerbuna, 12, 50009 Zaragoza, Spain; 4 Depto. Química Orgánica, Facultad de Ciencias, Universidad de Zaragoza, C/Pedro Cerbuna, 12, 50009 Zaragoza, Spain; 5 Department of Chemistry, UCIBIO, NOVA School of Science and Technology, Universidade NOVA de Lisboa, Quinta da Torre, 2829-516 Caparica, Portugal; 6 Associate Laboratory i4HB, Institute for Health and Bioeconomy, NOVA School of Science and Technology, Universidade NOVA de Lisboa, 2819-516 Caparica, Portugal

**Keywords:** Glycerol-derived ethers, Aquatic toxicology, Shrimp, Reactive oxygen species, Environmentally safe

## Abstract

In recent years, there has been extensive research within the scientific community on deep eutectic systems due to their remarkable versatility in solubilizing diverse substances and serving as effective solvents in catalytic processes. While initially regarded as non-toxic, a comprehensive toxicological assessment is essential to comprehend their behavior within organisms. In this study, seven distinct systems, composed of *N,N,N*-triethyl-*N*-(2,3-dihydroxypropyl)ammonium chloride (N00Cl) and glycerol-derived ethers with alkyl chains of varying lengths (100, 200, 3F00, 300, 3i00, and 400), in a 1:2 molar ratio were investigated for their aquatic toxicity in shrimp (*Palaemon varians*). The assessment involved analyzing oxidative stress biomarkers such as glutathione S-transferase, glutathione peroxidase, catalase, superoxide dismutase, total antioxidant capacity (TAC), and lipoperoxidation (MDA content). Results show an odd–even effect for LC_50_ values being **N00Cl-300**, the system showing higher values. Regarding oxidative stress, an imbalance between reactive oxygen species (ROS) and antioxidant capacity in the organisms has been observed, suggesting significant toxicity to shrimps due to the changes in oxidative stress biomarkers at high concentrations. However, at 100 mg/l all systems can be considered environmentally safe, and no negative impacts are expected on aquatic ecosystems.

**Supplementary Information:**

The online version contains supplementary material available at 10.1007/s11356-024-34983-3.

## Introduction

Eutectic systems are formed by combining substances that interact at the molecular level to lower the melting point (Chakraborty et al. 2021). In other words, they are combinations of two or more substances, which together have a lower melting point than the individual components (Abbott et al. 2003). Due to their unique physicochemical properties, these mixtures have attracted increasing interest in various industrial and technological areas. The applications of these deep eutectic systems (DES) are diverse and widely diversified: one of the most common uses is in the pharmaceutical industry, where they are used as solvents and vehicles for drug delivery (Chakraborty et al. 2021). They can also be found in the food industry (Mišan et al. 2020) as additives and texture improvers (Hayyan et al. 2012), as well as being used in other biological and chemical processes such as cryopreservation (Hornberger et al. 2021), biocatalysis (Xu et al. 2017), synthesis (Zhang et al. 2012; Yu et al. 2022), extraction (Duan et al. 2016), gas chromatography (Momotko et al. 2022).

Additionally, there has been growing interest in the antimicrobial and cytotoxic properties of DESs (Marchel et al. 2022). Such research highlights the potential of DESs in applications such as antimicrobial agents, which opens new avenues for their use but also raises concerns regarding their safety and environmental impact.

As the industrial and technological interest in these eutectic systems increases, it becomes crucial to understand and assess the potential risks to human and environmental health. Recent studies have questioned whether deep eutectic solvents might contribute to environmental harm, considering their volatility and potential to release harmful substances under certain conditions (Janjhi et al. 2023). Similarly, the possibility of cross-contamination of water bodies by DESs is another significant environmental issue. Hydrophobic deep eutectic solvents (HDESs), which are increasingly used as extractants for removing pollutants from water and wastewater, could pose a risk of introducing these solvents into aquatic systems, leading to contamination (Marchel et al. 2023). These concerns underscore the need for comprehensive environmental assessments of DESs, particularly as their industrial applications expand.

Ecotoxicological and toxicological testing are essential within the REACH framework (Garralaga et al. 2022b), In the European context, companies are required to perform (eco)toxicological testing, depending on the level of production, to ensure adequate risk management and to allow the placing of new chemicals substances on the market. These tests may vary depending on the quantity of the substance, its intended use, and the level of concern associated with its properties (acute toxicity, chronic toxicity, genotoxicity, carcinogenicity, etc.). Generally, conducting multiple aquatic assays, both in crustaceans and algae, fish, and aquatic plants, is a common practice to evaluate the environmental impact of the compounds under study. The aquatic toxicity of some of these mixtures has already been studied in marine biomodels such as *Daphnia magna* (Perales et al. 2017; Errazquin et al. 2021), *Aliivibrio* *fischeri* (Ventura et al. 2014; García et al. 2015; Garralaga et al. 2022a), or algae (Lapeña et al. 2021; Garralaga et al. 2022a). Their toxicological potential has also been investigated in fish (Juneidi et al. 2016; Lapeña et al. 2021) and other aquatic organisms such as shrimps (Hayyan et al. 2013b, a) and marine plants (Wen et al. 2015). *Palaemon varians* is an invertebrate aquatic organism used as a biomodel in aquatic toxicity studies (Jeliński et al. 2019). It is a species of shrimp that is particularly sensitive to chemical pollutants in water.

The use of this biomodel has numerous advantages: both collection and maintenance are relatively simple. It is a species on which physiological responses such as the concentration of antioxidant enzymes can be measured. Finally, it plays a crucial role in aquatic ecosystems by fulfilling indispensable functions within the food chain, such as the decomposition of organic material or the control of other marine populations (Saborowski et al. 2022). Oxidative stress results from an imbalance between the production of reactive oxygen species (ROS) and the antioxidant capacity of the organism. Increased ROS production in a species is indicative of cell damage and is therefore a quantifiable parameter in (eco)toxicity studies. Some of the most used antioxidant enzymes to quantify the antioxidant capacity of a substance are superoxide dismutase (SOD), catalase (CAT), and peroxidase. On the other hand, it is also common to find assays in which glutathione (intracellular antioxidant) levels are measured. Antioxidant enzyme studies provide a rapid and easily quantifiable method for assessing antioxidative capacity through the removal of products produced by oxidative stress. The information obtained from these studies makes it possible to study at a molecular and cellular level how a substance can affect a particular organism.

In order to understand the effects of eutectic mixtures on aquatic organisms and ecosystems, the ecotoxicity of seven DES consisting of *N*,*N*,*N*-triethyl-*N*-(2,3-dihydroxypropyl)ammonium chloride (N00Cl) as HBA and glycerol-derived ethers as (HBA) at different concentrations will be analyzed in this trial. In addition, biomarkers of oxidative stress (catalase, glutathione-S-transferase, glutathione peroxidase, superoxide dismutase, lipid peroxidation, and total antioxidant capacity) were measured to determine the biochemical impact of these substances on the *P. varians* biomodel.

These systems present interesting physicochemical properties and very low eco-toxicity (Perales et al. 2017); these glycerol-derived solvents are being used in replacing glycerol as the HBD component in eutectic solvents (Leal-Duaso et al. 2017b). These DES have successfully been applied to the design of recoverable homogeneous catalytic systems (Leal-Duaso et al. 2020, 2021) and are currently under study as solubilization media of bioactive compounds. Therefore, it is crucial to conduct ecotoxicological studies of these systems to assess potential residues that could be generated at an industrial level in the event of a spill into a river or effluent. The concentrations tested are high to understand the consequences of elevated levels in these systems.

## Materials and methods

### Preparation of DES

Sigma-Aldrich supplied glycidol, 3-chloropropane-1,2-diol, and triethylamine. Scharlab provided MeOH, EtOH, i PrOH, and potassium or sodium hydroxide. Alfa Aesar was the source for BuOH, 2,2,2-trifluoroethanol, phenol, 2-methoxyphenol, and choline chloride. The procedure for preparing deep eutectic systems (DES) has been detailed in prior descriptions (Leal-Duaso et al. 2017a, b). Briefly, DES were obtained by mixing *N*,*N*,*N*-triethyl-*N*-(2,3-dihydroxypropyl)ammonium chloride (N00Cl) as HBA and **100**, **200**, **3F00**, **300**, **3i00**, and **400** glycerol ethers as HBD in a 1:2 molar ratio. The mixtures were stirred in a glass vial at 70 °C until a transparent mixture was formed, after which they were cooled down to room temperature. Before each essay, all the systems were dried under vacuum for 24 h, due to the high hygroscopicity, before use.

### Exposure trials

Shrimps (*P. varians*), kindly provided by a local aquaculture (Ribeira das Enguias), served as the toxicological biomodel for testing deep eutectic systems (DES). Following a 24-h acclimatization period in laboratory conditions, the shrimps were allocated to 5-l aquariums equipped with a filtered seawater system. The exposure tests were performed with filtered seawater (VibrantSea, Seachem, USA) and salinity, 33 g/l. Throughout the experiment, pH (8 ± 0.1) and temperature (23.0 ± 0.1 °C) were carefully controlled. The shrimps experienced a 12-h light and 12-h dark photoperiod, along with continuous oxygenation (> 6 mg/l dissolved oxygen). For each experiment, a random selection of 5 shrimps (*n* = 30; 231.3 ± 121 mg) was exposed to five different concentrations (5000 mg/l, 2500 mg/l, 1000 mg/l, 500 mg/l, and 100 mg/l) of the chosen DES, distributed by 2-l polystyrene aquariums. The shrimps received daily feedings of commercial dry food (Sera brand), and laboratory parameters such as pH and temperature were monitored daily. Each experiment was conducted in duplicate over 7 days, with shrimp mortality recorded throughout the process.

Upon completing the experimental trial, the shrimps were placed in Eppendorf tubes (1.5 ml), weighed, and subsequently frozen at − 80 °C for preservation until further analysis of oxidative stress biomarkers.

### Biochemical essays

#### Sample treatment

The frozen shrimps were homogenized in 2 ml of PBS, and after grinding with the aid of a tissue homogenizer (Tissue Master 125, Omni, Kennesaw, GA, USA), the homogenate was centrifuged at 15,000 g for 10 min at 4 °C (VWR, CT 15RE, Hitachi Koki, Tokyo, Japan). The resulting supernatant was collected and frozen at − 80 °C for subsequent sample evaluations. To facilitate data analysis, samples were diluted (1/10 dilution) in phosphate saline buffer (PBS). Prior to conducting any enzymatic assays, protein quantification was performed using the Bradford method to normalize the biochemical results (Bradford 1976).

#### Glutathione S-transferase (GST)

The methodology used for assessing glutathione S-transferase (GST) activity (EC 2.5.1.18) was based on the procedure initially described by Habig et al. (Kato and Naito 1974)*.* It involves measuring the increase in absorbance at 340 nm, attributed to the formation of the conjugate between reduced glutathione (GSH) and 1-chloro-2,4-dinitrobenzene (cDNB). The method was adapted for a 96-well plate, where 20 µl of the centrifuged supernatant and 180 µl of the substrate solution (phosphate buffer saline with 100 mM cDNB and 200 mM GSH from Sigma-Aldrich) were added to each well. Enzymatic activity was monitored every minute for 6 min at 340 nm using a microplate reader (Synergy HTX, BioTek, Winooski, VT, USA). Results are expressed in nmol.min^−1^·mg^−1^ total cytosolic protein of the sample.

$$GST\;activity= \frac{(\Delta\;A340\;sample/min -\Delta\;A340\;blank/min) \times\;Vtotal(ml) \times\;dilution\;}{0.0053\;\times Vsample\;(ml)}$$

Equation 1 shows the determination of glutathione-S-transferase activity.

#### Glutathione peroxidase (GPx)

The glutathione peroxidase (GPx) activity (EC 1.11.1.9) is based on the methodology employed by Lawrence and Burk (1976).

In brief, 20 µl of each previously described sample (refer to the “Sample treatment” section) was combined with a 96-well plate containing 120 µl of a buffer solution (comprising 5 mM EDTA and 50 mM phosphate buffer) and 50 µl of a co-substrate mixture, consisting of 1 mM nicotinamide adenine dinucleotide phosphate (NADPH, Sigma-Aldrich, Germany), 4 mM reduced glutathione (GSH, Sigma-Aldrich, Germany), 4 U/ml glutathione reductase (GSSG-reductase, Sigma, Germany), 1 mM nicotinamide adenine dinucleotide phosphate (NADPH, Sigma-Aldrich, Germany), and 4 mM sodium azide (Sigma-Aldrich, Germany). The initiation of the enzymatic reaction involved adding 20 µl of 15 mM hydroperoxide cumene (Sigma-Aldrich, Germany). Enzymatic activity, expressed as nmol.min^−1^·mg^−1^ total protein, was determined by measuring the decrease in absorption per minute at 340 nm using a microplate reader (Synergy HTX, BioTek, Winooski, VT, USA), which is proportional to the reduction of β-NADPH.

$$GPx\;activity= \frac{{~}^{Abs 340}\!\left/ \!{~}_{\mathit{min}\times V \left(ml\right) \times dilution}\right.}{0.00373 \times\;Vsample (ml)}$$

Equation 2 shows the glutathione peroxidase activity determination.

#### Catalase (CAT)

The method involves assessing catalase activity (EC 1.11.1.6) by the colorimetric measurement of the formaldehyde produced by the catalase reaction. The methodology was adapted to a 96-well plate from Johansson and Håkan Borg (1988). To assess catalase activity in each sample, a calibration curve ranging from 0 to 75 µM formaldehyde (Sigma-Aldrich, Germany) was generated. In a nutshell, 70 µl of shrimp homogenate supernatant was combined with 50 µl of a buffer solution (100 mM potassium phosphate, pH 7.0) and 30 µl of methanol (Scharlau, Spain) in each well of a 96-well plate. Subsequently, 20 µl of 0.035 M hydrogen peroxide (Sigma-Aldrich, Germany) was added to initiate the enzymatic reaction. Following 20 min of plate incubation under agitation (Fisherbrand microplate shaking), 30 µl of 10 M potassium hydroxide (Chem-Lab, Belgium) and 30 µl of Purpald solution (34.2 M in 0.5 M HCl, Sigma-Aldrich, Germany) were added. The plate underwent a 10-min incubation with a light cover and constant shaking. Finally, 10 µl of potassium periodate (65.2 mM, Chem-Lab, Belgium) was added to halt the enzymatic reaction. After a 5-min incubation, the plate was spectrophotometrically read at 540 nm using a plate reader (Synergy HTX, BioTek, Winooski, VT, USA). Catalase activity is determined by the formaldehyde produced in each sample, and the results are expressed by normalizing to the total protein mass (nmol.min^−1^·mg^−1^ total protein).

#### Superoxide dismutase (SOD)

The assay for this enzyme (SOD, EC 1.15.1.1) involves the inhibition of nitroblue tetrazolium reduction (NBT) and was performed according to Sun et al. (1988) after being adapted to a 96-well plate. SOD facilitates the dismutation of radicals (O_2_^−^) in competition with NBT. In summary, each well of a 96-well plate received 10 µl of 3 mM xanthine (Sigma-Aldrich, Germany), 200 µl of phosphate buffer (50 mM; pH 8.0), 10 µl of 0.075 mM NBT (Sigma-Aldrich, Germany), 10 µl of 3 mM EDTA, and 10 µl of the sample. Subsequently, 10 µl of 10 U/ml xanthine oxidase (Sigma-Aldrich, Germany) was added to each well. The absorbance was measured at 535 nm every 2 min for a total duration of 10 min using a plate reader (Synergy HTX, BioTek, Winooski, VT, USA). Negative controls (distilled water and PBS) were included in the experiment to represent the maximum increase in absorbance. The percentage inhibition per minute reflects SOD activity and is expressed as % inhibition per total protein concentration.

$$\%\;SOD\;inhibition=\frac{ (\Delta\;A560\;negative\;control/min\;-\;\Delta A560\;sample/min) }{\Delta \;A560\;negative\;control/min}$$

Equation 3 shows the superoxide dismutase inhibition (%).

#### Total antioxidant capacity (TAC)

This spectrophotometric method relies on the reduction of the 2,2′-azino-bis-3 ethylbenzothiazoline-6-sulfonic acid (ABTS) cation radical, following the method outlined by Kambayashi et al. (2009). Trolox served as the standard antioxidant (ranging from 0 to 0.33 mM) to build a calibration curve. In summary, each well of a 96-well microplate received 10 µl of 90 µM myoglobin (Sigma, Germany), 150 µl of 600 µM ABTS (Alfa Aesar, Germany), and 10 µl of the sample. The reactions were initiated by adding 40 µl of hydrogen peroxide (500 µM, Sigma-Aldrich, Germany). After a 10-min incubation period, absorbance was measured at 415 nm using a microplate reader (Synergy HTX, BioTek, Winooski, VT, USA). The obtained results are expressed normalized to the protein mass of each sample (nmol·mg^−1^ total protein).

#### Lipid peroxidation

The thiobarbituric acid (TBA) method followed the protocol established by Uchiyama and Mihara (1978). The assay mixture, prepared in 2-ml microtubes, involved combining 5 µl of each sample (supernatant after centrifugation), 93.5 µl of trichloroacetic acid (20%, Panreac, Spain), 93.5 µl of thiobarbituric acid (Sigma-Aldrich, Germany), 12.5 µl of SDS (8.1%, Sigma-Aldrich, Germany), 45 µl of a phosphate buffer (pH 7–7.4), and 50.5 µl of MQ-grade ultrapure water. After vigorous agitation, the microtubes were subjected to boiling water (100 °C for 5 min), after puncturing caps, to initiate the reaction and promptly cooled on ice. Subsequently, 62.5 µl of MQ-grade ultrapure water was added. Then, the microtubes were thoroughly shaken, and 150 µl of each microtube’s content was transferred to each well in a 96-well microplate. The MDA content of the samples was determined by measuring absorbance at 530 nm using a microplate reader (Synergy HTX, BioTek, Winooski, VT, USA). A calibration curve with malondialdehyde bis(dimethylacetal) (MDA, Merck) standards facilitated the quantification of lipid peroxidation (0–0.1 µM). The results were expressed as nmol·mg^−1^ total protein.

### Statistical analysis

Statistical analysis was performed using Prism 9.0 (GraphPad Software). All results are expressed as mean ± standard deviation (SD). Comparisons were carried out through the Kruskal–Wallis test or one-way ANOVA. Additionally, Dunnett’s multiple comparisons test was used to analyze each experiment.

## Results and discussion

### Mortality rate

LC_50_ values, along with their corresponding standard deviations, are compiled in Table 1. Moreover, EC_50_ values for alternative aquatic biomodels (*A. fischeri* and *R. subcapitata*) are also provided, enabling a comparison of the behavior across three different species used as biological models in toxicity studies. Additionally, the mortality rate of shrimps exposed to the studied DES was evaluated. Figure S1 shows the normalized mortality rate of the studied DES at various concentrations. There is a positive correlation between shrimp mortality and exposure concentration. In all compounds, there is an increase in the number of deaths with increasing concentration.

**Table 1:** Values of *P. varians*, LC_50_ (mg/l) and values of EC_50_ (mg/l) of studies with *A. fischeri* and *R. subcapitata* exposed to DES

DES	LD_50_ (mg/l)	EC_50 (_mg/l)	
	*P. varians*	*A. fischeri* [16]	*R. subcapitata* [16]
N00Cl-000	3835 ± 994	83277 ± 4282	7015 ± 170
N00Cl-100	4434 ± 1716	93192 ± 4487	11,423 ± 924
N00Cl-200	2924 ± 510	8089 ± 128	7343 ± 567
N00Cl-300	5002 ± 315	16976 ± 2766	8087 ± 523
N00Cl-400	1052 ± 248	3446 ± 1132	5828 ± 666
N00Cl-3F00	1157 ± 284	34957 ± 4525	12,560 ± 196
N00Cl-3i00	2015 ± 1026	24754 ± 1205	9597 ± 1205

It is important to note that in some concentrations tested no shrimp survived. For instance, **N00Cl-000**, **N00Cl-100**, **N00Cl-200**, and **N00Cl-300 systems** showed surviving shrimp were observed at all concentrations tested; however, in the case of N**00Cl-400**, **N00Cl-3F00**, and **N00Cl-3i00**, no surviving animals were observed at 2500 and 5000 mg/l.

When the EC_50_ data obtained are analyzed, it is observed that in the systems with even chains, the EC_50_ values decrease as the chain length increases being the less toxic **N00Cl-000** followed by **N00Cl-200** and **N00Cl-400**. However, this trend is not observed in the case of the odd chain, where the toxicity is higher for **N00Cl-100** than **N00Cl-300**.

Additionally, the toxicity of the **N00Cl-300** system is slightly higher than for the **N00Cl-3i00**, showing that when ramifications are included in the HBD alkyl chain, an increase of the EC_50_ value has been observed and therefore a decrease in DES toxicity.

Furthermore, the influence of the presence of fluorine atoms can be observed when comparing **N00Cl-200** and **N00Cl-3F00** mixtures. When fluorine atoms are incorporated into the structure of the HBD component, an increase in toxicity is observed; this trend is only observed for the *P. varians* biomodel. For the other two aquatic biomodels presented in Table 1, it can be observed that the EC_50_ of compound **N00Cl-3F00** is higher, in both cases, than the compound **N00Cl-200**, indicating lower toxicity. The impact of fluoride on algae and bacteria can vary, either inhibiting or promoting their growth depending on factors such as fluoride concentration, duration of exposure, and the specific species involved. Aquatic plants have shown potential in effectively removing fluoride from polluted water in both controlled laboratory settings and natural environments; however, in aquatic animals, fluoride tends to accumulate in the exoskeletons of invertebrates and in the bone tissues of fish (Camargo 2003).

For all the studied systems, a concentration-dependent toxicity has been obtained. This relationship has been studied in the field of toxicology and for other DES (Zwart et al. 1990; Inayat et al. 2022).

Regarding LC_50_ values, there is a clear trend between toxicity and increased alkyl chain in HBD for even chain DES. This trend has been observed in the aquatic bioindicators, *R. subcapitata,* but not for *A. fischeri*. Nevertheless, this trend is not observed for the case of the odd ones, where the toxicity decreases as the alkyl chain increases. The “even–odd effect” in the toxicity of alkyl chains has been already reported and refers to the observation that organic compounds with an even number of carbon atoms tend to exhibit different toxic properties compared to those with an odd number (Adachi et al. 1995). A proposed explanation for this effect is based on the physicochemical properties and molecular structure of the alkyl chains. It is suggested that alkyl chains with an even length may exhibit greater symmetry and more efficient packing in cell membranes or at biological binding sites compared to odd-length chains. This increased symmetry can affect transport properties across biological membranes and affinity for biological receptors (Zein and Winter 2000; De Meyer et al. 2008).

In addition to these comparisons, it is also necessary to analyze how mortality changed when comparing the **N00Cl-200** and **N00Cl-3F00** systems. The bioindicators *A. fischeri* and *R. subcapitata* show that toxicity decreases by including carbon atoms in the chemical structure; however, this trend is contrary to that observed in shrimp. When comparing **N00Cl-300** and **N00Cl-3i00** systems, the same trend observed in other bioindicators is noted: the presence of radicals decreases toxicity in different biomodels. This may be related, as mentioned in other articles (García et al. 2015), to the increased difficulty in crossing biological barriers due to branching and molecular weight increase of the DES. However, this effect is less pronounced in the case of the *P. varians* biomodel compared to the bacterial model and the algae.

From the three aquatic biomodels tested, it can be observed that, although toxicity initiates at very high concentrations and according to the Passino and Smith Classification (PSC) (Passino and Smith 1987), these systems could be categorized as non-hazardous for aquatic medium; however, *P. varians* exhibits a higher sensitivity to these systems. This is particularly highlighted by the introduction of fluorides within the eutectic system, where the difference in EC_50_/LD_50_ is up to 30 times for *A. fischeri* and 10 times for *R. subcapitata*. This could be due to these biomodels representing different trophic levels, with shrimps being a much more complex biomodel than bacteria and algae. Although ecotoxicological data for these compounds in *P. varians* are not available, Perales et al. determined the ecotoxicity of the HBD compounds (glycerol ethers) in the crustacean *Daphnia magna* (Perales et al. 2017). The components forming the HBD appear to individually present lower toxicity than the eutectic mixture. However, we should not overlook the difference in biomodels and the fact that there is no toxicity data for the HBA (N00Cl), making it impossible to determine if synergies in toxicity exist.

In general, we observe that for all biomodels, although the sensitivity to systems may not be the same, an even–odd effect in toxicity is evident. Additionally, as seen in most toxicological studies conducted on eutectic mixtures, modification of the HBD contributes to changes not only in the physicochemical properties of the mixture but also in its ecotoxicological properties. These compounds have only been previously studied in two other aquatic biomodels, making it still challenging to establish a structure–toxicity relationship (Garralaga et al. 2022a).

However, it is crucial to note the distinctiveness between this species, comprising bacteria and microalgae, and shrimp, as they may exhibit varying responses to DES exposure. While algae and bacteria have been utilized in assessing DES toxicity, they serve as unicellular models, whereas shrimp represent a more biologically complex organism (Brown et al. 2020; Saborowski et al. 2022). Moreover, the parameters such as pH, temperature, and culture media differ between the *A. fischeri* and *R. subcapitata* tests and shrimp tests, potentially influencing DES interactions with the medium and organisms, thus yielding different effects on the assayed models (El Achkar et al. 2019).

Numerous studies have investigated the impact of acidity/alkalinity and water content on DES physicochemical properties and behavior. For instance, Jançiková et al. noted a linear decrease in DES pH with rising temperature. The type of hydrogen bond donors significantly affects DES acidity, while water content influences properties like polarity and solubilization capacity, as corroborated by Skulcova et al. (Skulcova et al. 2019)*.* Regarding system bioavailability, besides pH, considerations such as chemical structure, ionic strength, and co-solvent presence play crucial roles, as highlighted by Smulek et al. in their bacterial studies, with potential extrapolation to other organisms (Smułek and Kaczorek 2022).

In the case of shrimp, it is noteworthy that this biomodel readily adapts to environmental changes, particularly in salt concentrations. A study by Missionario et al. demonstrated their adeptness in hyper- and hypo-osmoregulation, which is pivotal for maintaining cellular function across varying salinities (Missionário et al. 2023).

### Glutathione S-transferase (GST)

**Fig. 1:**
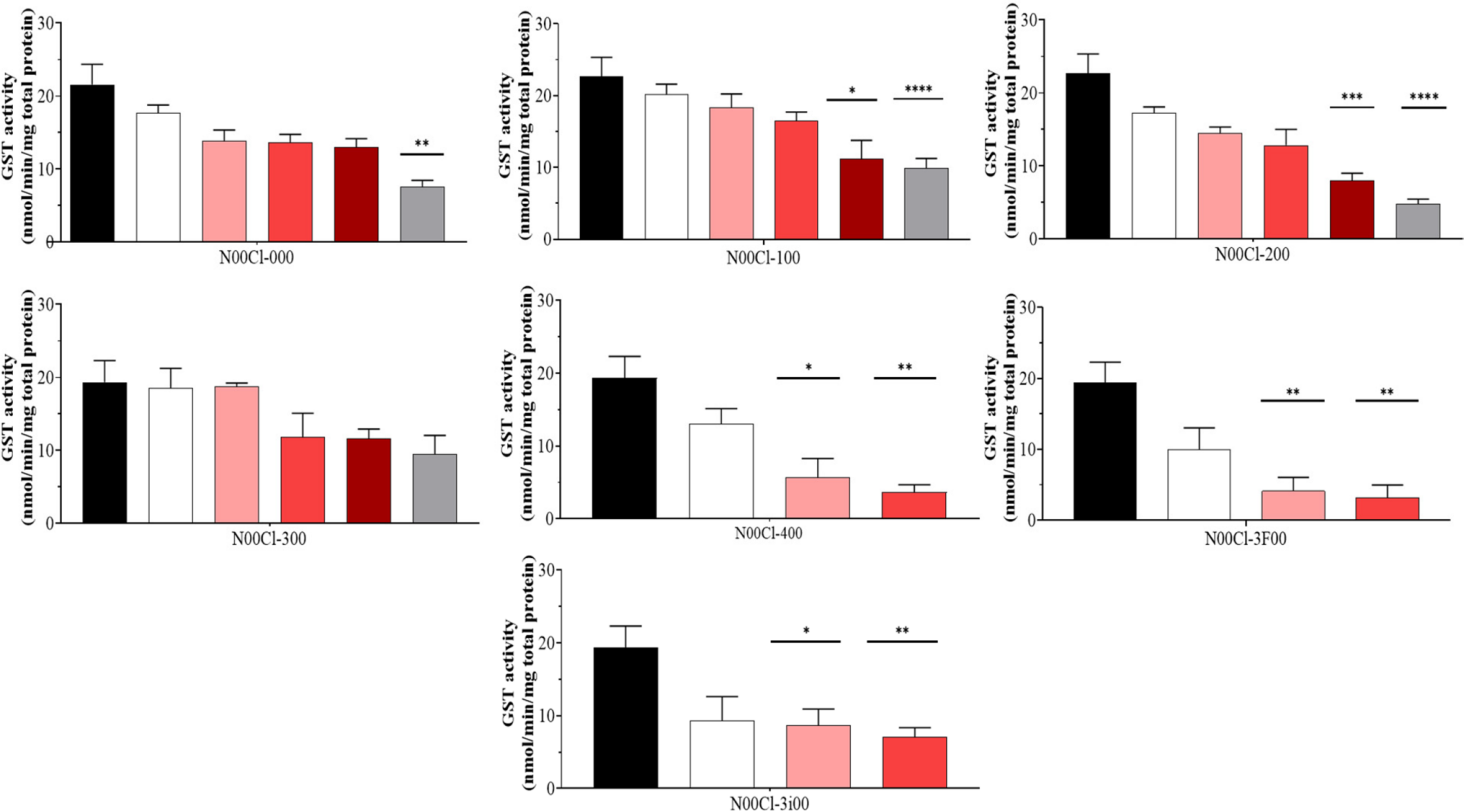
Glutathione-S-transferase (GST) activity (mean ± SD) in *P. varians.* The concentrations tested were 0 mg/l (black bar), 100 mg/l (white bar), 500 mg/l (rose bar), 1000 mg/l (red bar), 2500 mg/l (dark red bar), and 5000 mg/l (grey bar)

In Fig. 1, glutathione S-transferase activity, at different DES concentrations, is depicted. In general, in all cases, it is observed that the activity decreases as the concentration of DES used increases.

It is observed that for **N00Cl-000**, there are no significant differences with the control up to concentrations of 2500 mg/l, but in the case of **N00Cl-100**, this trend is observed up to concentrations of 1000 mg/l. The **N00Cl-300** system is the one that shows no differences with respect to the control at any concentration except 2500 mg/l. Finally, in the **N00Cl-200**, **N00Cl-400**, **N00Cl-3F00**, and **N00Cl-3i00** systems, it is observed that the only concentration that does not show differences with respect to the control is 100 mg/l, indicating that these systems are probably not as safe as they generate modifications in the enzyme.

The Spearman’s correlation coefficient was determined, revealing a significant negative correlation (*r* < 0; *p* < 0.05) for **N00Cl-000**, **N00Cl-100**, and **N00Cl-200** systems across all other enzymatic assays conducted (GPx, CAT, TAC, LPO, and SOD). In the case of **N00Cl-300**, this negative correlation became significant for TAC assays (*p* = 0.04; *r* =  − 0.46), LPO assays (*p* = 0.004; *r* =  − 0.61), and SOD assays (*p* = 0.03; *r* =  − 0.63). A similar trend was observed for **N00Cl-400**, but with GPx (*p* = 0.005; *r* =  − 0.71), CAT (*p* = 0.009; *r* =  − 0.68), and LPO (*p* = 0.015; *r* =  − 0.64) assays. Finally, for **N00Cl-3F00** and **N00Cl-3i00** systems, this correlation occurred with GPx (*p* = 0.004; *r* =  − 0.67 and *p* = 0.01; *r* =  − 0.61, respectively) and LPO (*p* = 0.02; *r* =  − 0.62 and *p* = 0.03; *r* =  − 0.58, respectively). These results are shown in Figure S2 of the Supplementary material.

### Glutathione peroxidase (GPx)

**Fig. 2:**
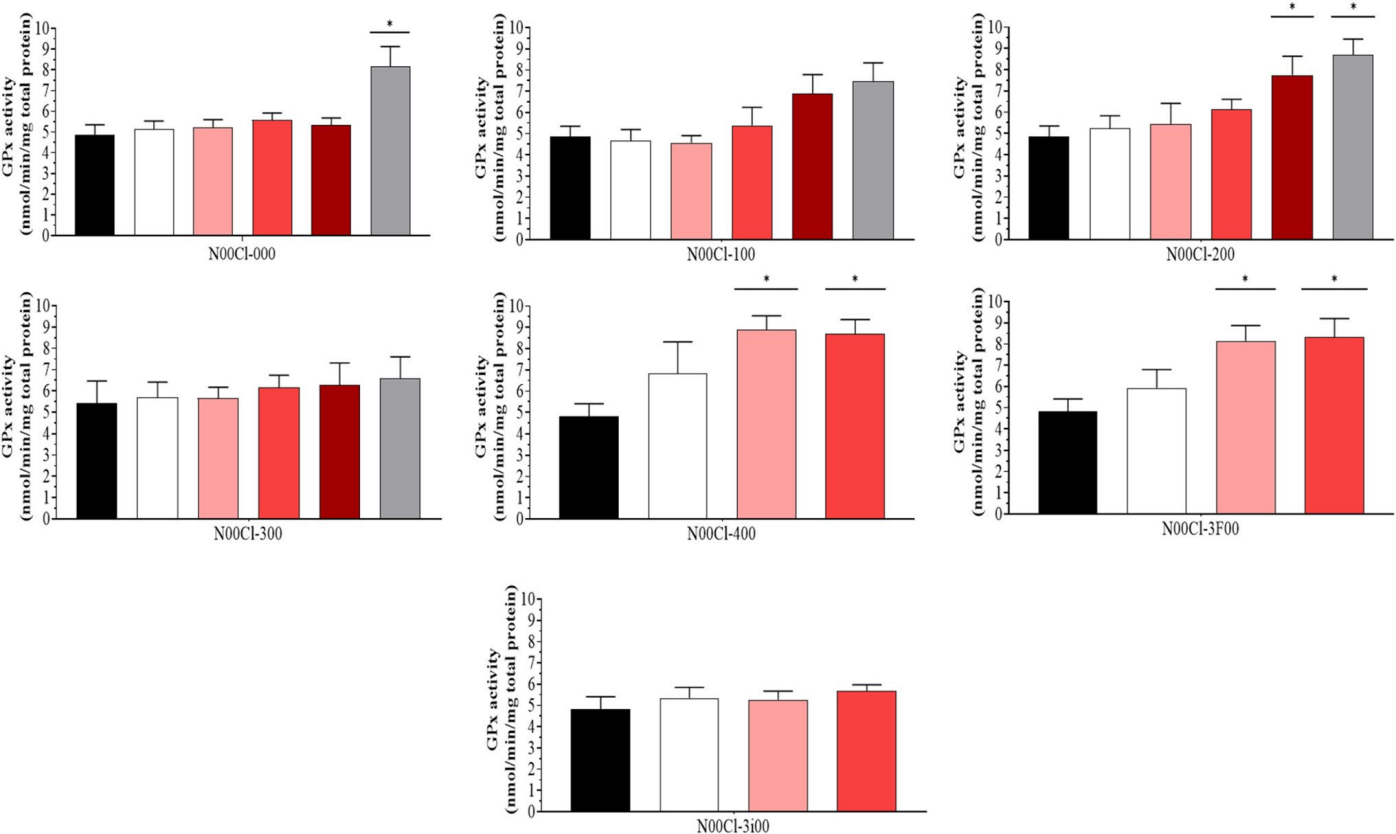
Glutathione peroxidase (GPx) activity (mean ± SD) in *P. varians.* The concentrations tested were 0 mg/l (black bar), 100 mg/l (white bar), 500 mg/l (rose bar), 1000 mg/l (red bar), 2500 mg/l (dark red bar), and 5000 mg/l (grey bar)

The average concentrations of GPx activity in shrimp are shown in Fig. 2. The results indicate that for **N00Cl-100** and **N00Cl-300** systems, no significant differences were observed at any concentration, so the maximum exposure concentration was 5000 mg/l. However, for **N00Cl-200**, the concentration increases at 1000 mg/l. For the **N00Cl-400** and **N00Cl-3F00** systems, no significant differences with respect to the control were observed in the case of 100 mg/l, and finally, for **N00Cl-3i00**, no significant differences with respect to the control were observed in any case, so the enzymatic activity was not altered with respect to the control, not even at the highest concentration at which the shrimps survived (1000 mg/l) for this DES. Systems **N00Cl -200**, **N00Cl-400**, and **N00Cl-3F00** should be used in moderation, as it was observed that there were significant differences with the control, thus modifying the enzymatic activity of GPx.

For the **N00Cl-100** and **N00Cl-300** systems, the highest concentration of GST activity has been found at 5000 mg/l (7.48 nmol/min/mg total protein for **N00Cl-100** and 6.58 nmol/min/mg total protein for **N00Cl-300**, respectively), whereas the lowest concentration was measured at 100 mg/l (5.12 nmol/min/mg total protein and 5.64 nmol/min/mg total protein, respectively).

For GPx, as previously mentioned, a significant negative correlation is observed for all systems except for **N00Cl-300** in GST. On the other hand, both **N00Cl-000** and **N00Cl-400** show a significant increasing trend, such that an increase in GST activity leads to a significant increase in CAT activity (*p* = 0.01; *r* = 0.49 and *p* = 0.02; *r* = 0.63, respectively). We only observe a significant positive correlation for the lipoperoxidative activity (LPO) in **N00Cl-3i00** (*p* = 0.001; *r* = 0.802). **N00Cl-100** and **N00Cl-200** systems share the same positive correlation with CAT (*p* = 8.42e − 005; *r* = 0.68 and *p* = 0.001; *r* = 0.59, respectively) and TAC (*p* = 0.002; *r* = 0.58 and *p* = 2.89e − 004; *r* = 0.64, respectively).

### Catalase (CAT)

**Fig. 3:**
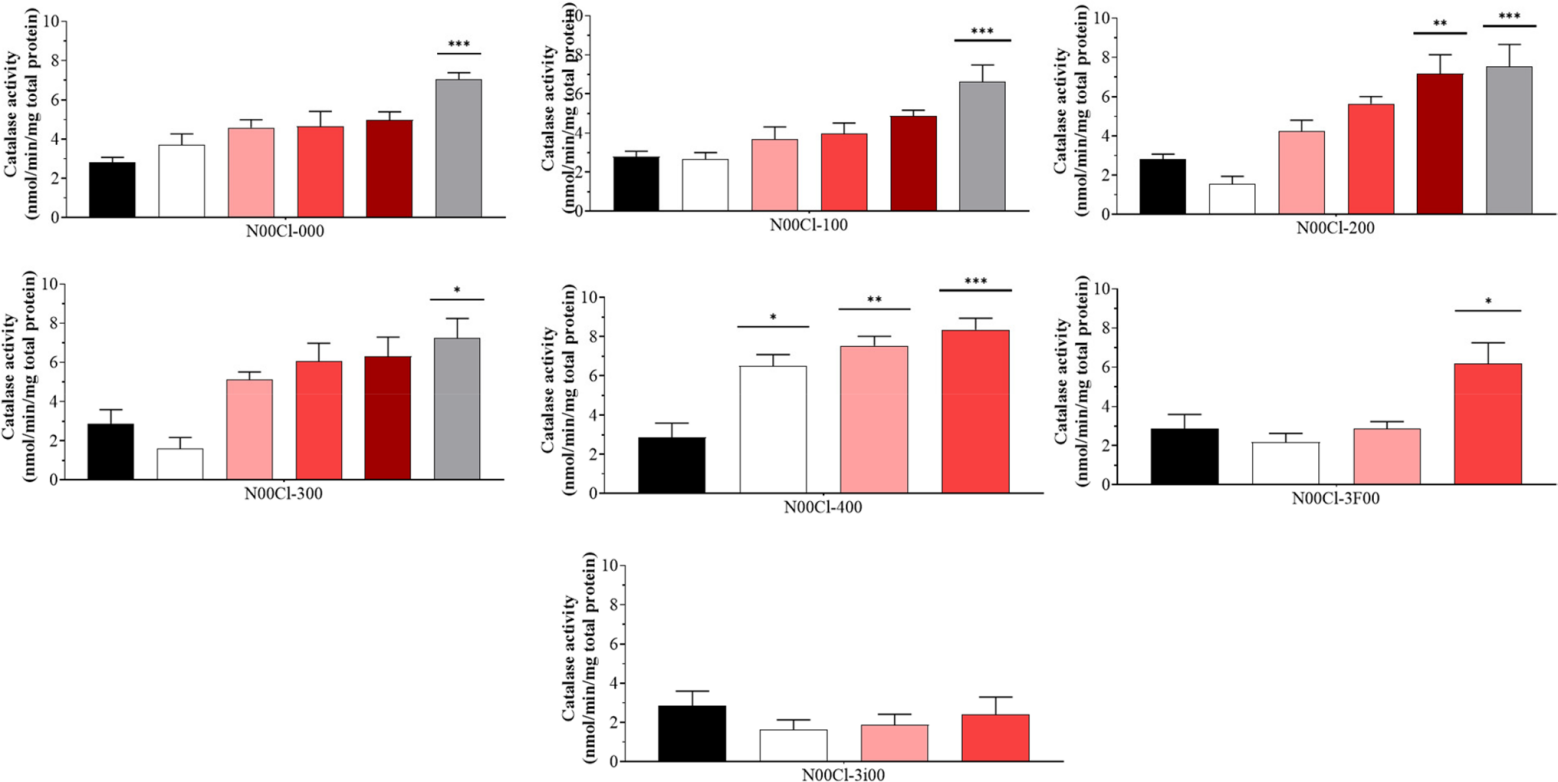
Catalase activity (mean ± SD) in *P. varians.* The concentrations tested were 0 mg/l (black bar), 100 mg/l (white bar), 500 mg/l (rose bar), 1000 mg/l (red bar), 2500 mg/l (dark red bar), and 5000 mg/l (grey bar)

In Fig. 3, the concentration of catalase activities in the cytosol is presented. When analyzing the figures, the data obtained is superior to the control, but when analyzing the systems separately, it can be seen that in the case of the **N00Cl-000**, **N00Cl-100**, and **N00Cl-300**, only the concentration of 5000 mg/l causes significant changes in enzymatic activity. For **N00Cl-200**, in addition to the 5000 mg/l concentration, the 2500 mg/l concentration also causes significant modifications. Note that in the case of **N00Cl-400**, all concentrations tested show significant differences, indicating that this system should be used with caution. Finally, **N00Cl-3F00** shows significant differences at concentrations of 1000 mg/l, and **N00Cl-3i00** shows no differences and very low enzymatic activity.

In this case, a significant positive correlation is observed between catalase activity and GPx, TAC, LPO, and SOD for **N00Cl-000**, **N00Cl-100**, and **N00Cl-200**. **N00Cl-300** exhibits the same positive correlation, but only for TAC (*p* = 0.015; *r* = 0.57) and SOD (*p* = 2.2e − 005; *r* = 0.814). On the other hand, no correlation is observed with other enzymatic assays for **N00Cl-3i00**, while both **N00Cl-400** and **N00Cl-3F00** positively correlate with lipid peroxidation, such that an increase in catalase activity corresponds to an increase in lipid peroxidation (*p* = 0.02; *r* = 0.518 and *p* = 0.01; *r* = 0.654, respectively).

### Superoxide dismutase (SOD)

**Fig. 4:**
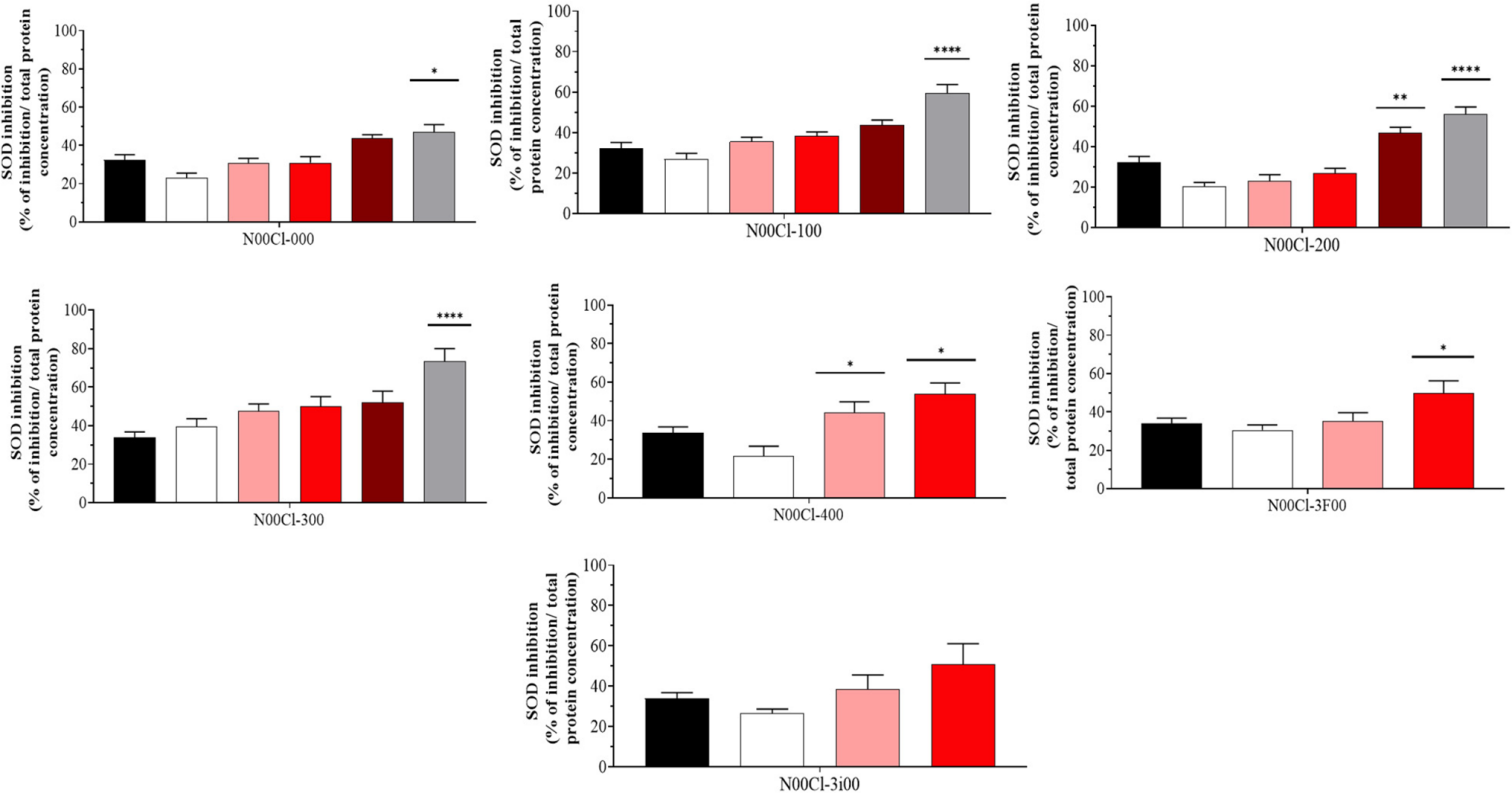
Superoxide dismutase activity as a percentage of inhibition (mean ± SD) in *P. varians.* The concentrations tested were 0 mg/l (black bar), 100 mg/l (white bar), 500 mg/l (rose bar), 1000 mg/l (red bar), 2500 mg/l (dark red bar), and 5000 mg/l (grey bar)

The superoxide dismutase activity (% of inhibition) is shown in Fig. 4. In this case, the SOD increases according to DES concentrations. Higher values are found for **N00Cl-000** (48.2%), **N00Cl-100** (59.6%), and **N00Cl-300** (73.4%) at 5000 mg/l. The highest observed SOD for **N00Cl-200** occurs at 2500 mg/l (46.9%) while for **N00Cl-400 and N00Cl-3i00**, the highest level is expressed at 500 mg/l (44.1%) and 1000 mg/l (35.5%), respectively. When fluorine atoms are introduced in the HBD, the inhibition of the enzyme decreases. Lower values for **N00Cl-3i00** were found when it was compared to **N00Cl-300**.

When determining the Spearman correlation coefficient (Supplementary Material 1), it was observed that the increase in SOD corresponded also to an increase in GPx, CAT, TAC, and LPO activity for **N00Cl-100** and **N00Cl-200** systems and **N00Cl-300**, indicating a significant positive correlation (*p* < 0.05; *r* > 0). For **N00Cl-000** and **N00Cl-400**, this positive correlation occurs between SOD and TAC and LPO activity (*p* = 4.98e − 007; *r* = 0.79 and *p* = 0.001; *r* = 0.56 for **N00Cl-000** and *p* = 0.02; *r* = 0.53 and *p* = 0.013; *r* = 0.57, respectively), while in the case of **N00Cl-3i00**, no significant correlation is observed.

### Total antioxidant capacity (TAC)

**Fig. 5:**
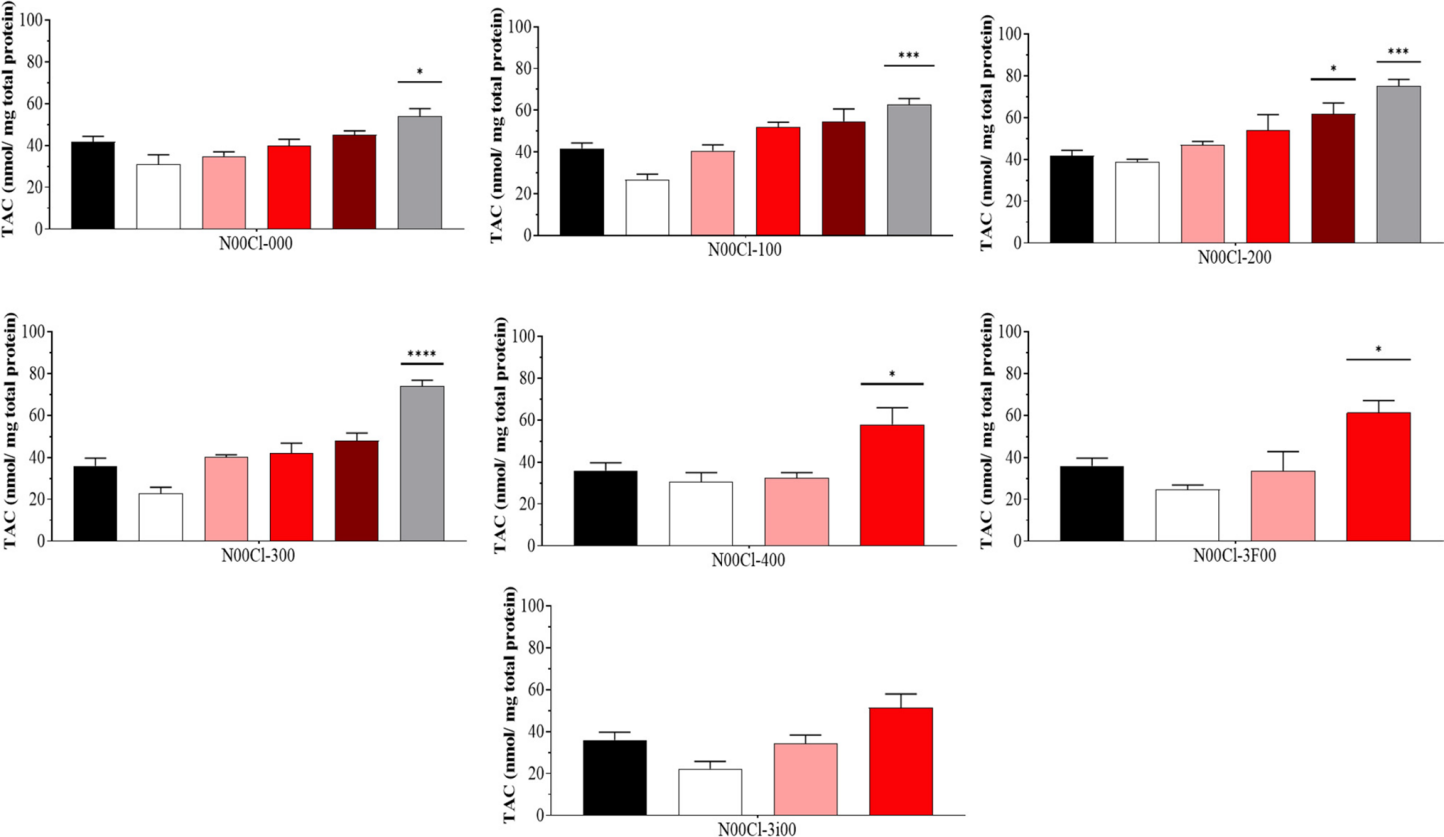
Total antioxidant capacity (mean ± SD) in *P. varians.* The concentrations tested were 0 mg/l (black bar), 100 mg/l (white bar), 500 mg/l (rose bar), 1000 mg/l (red bar), 2500 mg/l (dark red bar), and 5000 mg/l (grey bar)

The average concentration of TAC results in shrimps is presented in Fig. 5. For **N00Cl-000**, **N00Cl-100**, **N00Cl-200**, and **N00Cl-300**, it is observed that the TAC increases as the DES concentration increases, obtaining values higher than the control. On the contrary, in **N00Cl-400**, **N00Cl-3F00**, and **N00Cl-3i00**, some of the TAC values obtained are lower than those presented by the control. The highest value of TAC is obtained for **N00Cl-200** at 5000 mg/l (75.13 nmol/mg of total protein).

When comparing the **N00Cl-200** and **N00Cl-3F00** systems, it is observed that somewhat higher values are obtained in the system that does not contain fluorine atoms. On the contrary, a significant difference is observed between **N00Cl-30**0 and **N00Cl-3i00**, presenting much lower TAC values for the system containing isopropyl in the HBD.

As in all previous cases, there is a positive correlation for some enzymatic tests in certain DES. For example, we observe a significant positive correlation between CAT and GPX, CAT, LPO, and SOD for **N00Cl-100**, **N00Cl-200**, and **N00Cl-300** systems. Conversely, **N00Cl-000** shows a correlation only with LPO and SOD. Finally, **N00Cl-3F00** and **N00Cl-3i00** exhibit this positive correlation only between TAC and CAT activity and between SOD activity, respectively.

### Lipid peroxidation

**Fig. 6:**
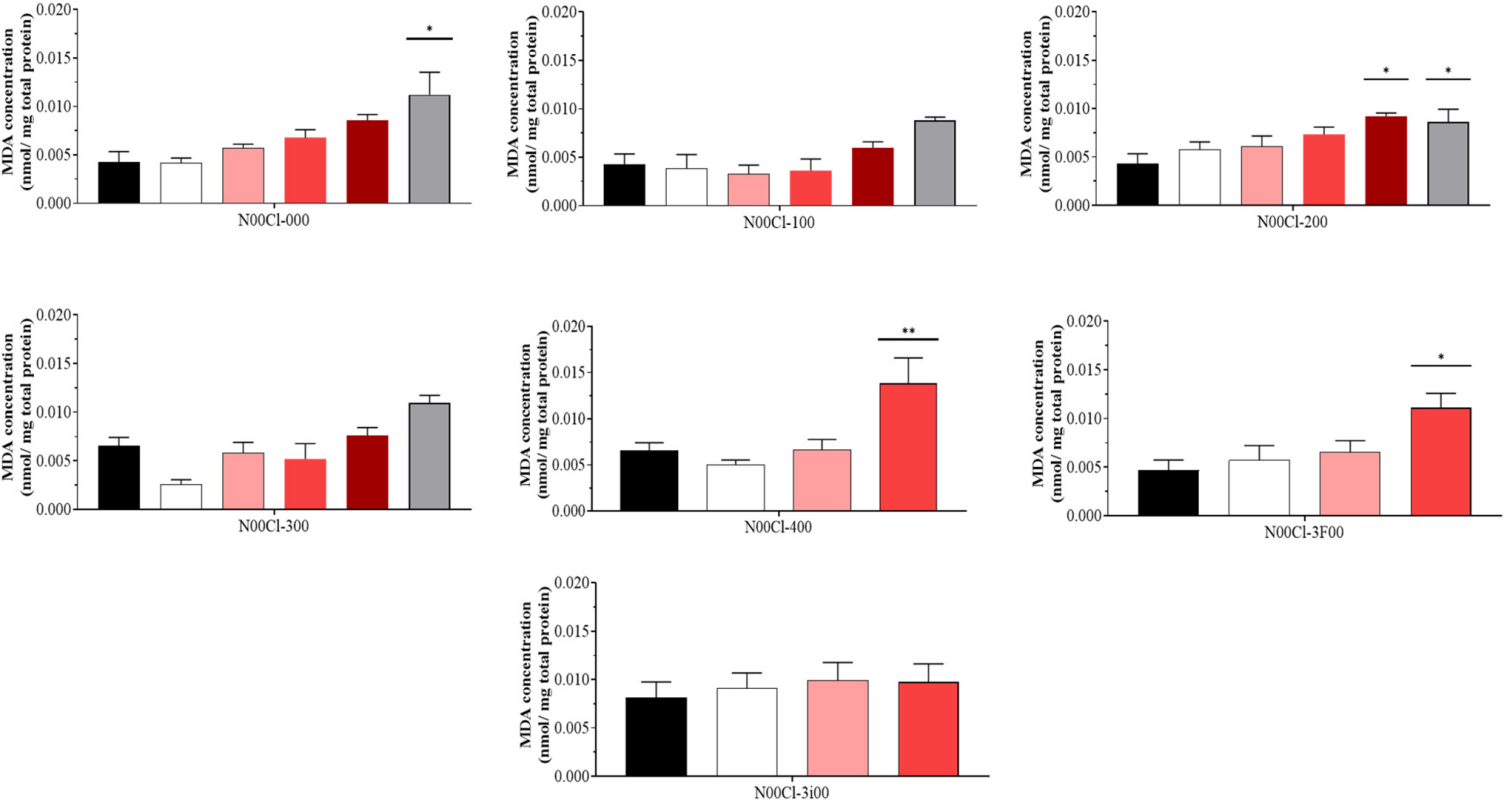
Lipid peroxidation (mean ± SD) in *P. varians.* The concentrations tested were 0 mg/l (black bar), 100 mg/l (white bar), 500 mg/l (rose bar), 1000 mg/l (red bar), 2500 mg/l (dark red bar), and 5000 mg/l (grey bar)

The average MDA concentrations obtained in the shrimps are presented in Fig. 6. It is observed that the general tendency is that the concentration of MDA increases as the concentration of DES increases. Usually, the values obtained are higher than those of the control, except for some cases at the lowest tested concentration (100 mg/l). In this case, **N00Cl-100**, **N00Cl-300**, and **N00Cl-3i00** systems do not present significant differences, indicating no changes compared to the control when shrimps are exposed to these DES. Conversely, the effects of lipid peroxidation become significant at very high concentrations in **N00Cl-000** (5000 mg/l) and **N00Cl-200** (2500 mg/l). For **N00Cl-400** and **N00Cl-3F00**, lipid peroxidative activity increases starting from 500 mg/l in both cases (0.014 and 0.009 nmol/mg total protein, respectively).

For most of the DES, **N00Cl-000**, **N00Cl-100**, **N00Cl-200**, **N00Cl-300**, and **N00Cl-400**, there is a positive correlation between lipid peroxidation and the enzymatic activity of CAT, SOD, and TAC. In the case of the **N00Cl-3F00** system, this correlation only occurs significantly with CAT (*p* = 0.045, *r* = 0.53), and for the compound **N00Cl-3i00**, we observe that it only occurs with GPx (*p* = 0.001, *r* = 0.802).

### Oxidative stress of deep eutectic systems

In this study, there is a lack of existing literature on the antioxidant activity of *P. varians* exposed to the studied DES. Several studies have explored the oxidative behavior of various DES across different models. For instance, Rasodevic et al. investigated the toxicological effects of eutectic systems formed by choline chloride (HBA) and oxalic acid in fresh wheat leaves, noting that high concentrations of ChCl:OA inhibited several enzymes, disrupting the antioxidant balance and leading to altered photosynthetic function (Radošević et al. 2014). Another study focused on a DES made from choline chloride and urea, which induced oxidative stress in mice kidneys, resulting in decreased catalase activity and increased malondialdehyde levels compared to the control group, indicating potential negative health impacts (Jung et al. 2021). Ferreira et al. analyzed the antioxidant profile of DES formed with citric acid:trehalose:water in *Danio rerio*, revealing an imbalance in the antioxidant system, with significant differences in enzymes such as CAT, GPx, GST, TAC, SOD, and MDA content compared to the control group (Ferreira et al. 2022). Additionally, enzymatic assays in systems composed of betaine:sorbitol:water and betaine:glycerol showed varied responses, with the betaine:glycerol system activating an antioxidant response surpassing the control, while the betaine:sorbitol:water system inhibited some enzymes like SOD while others showed increased activity at higher concentrations compared to the control (Ferreira et al. 2023).

Some assays analyze the oxidative profile of certain DES in different cell lines. Hayyan et al. studied the antioxidant profile of ammonium DES formed with different HBDs (glycerol (Gl), ethylene glycol (EG), triethylene glycol (TEG), and urea (U) using the ORAC assay. The results showed that DESs exhibit very low antioxidant activity compared to quercetin, indicating that these DESs cannot act as free radical scavengers (Hayyan et al. 2015). Another study evaluates the antioxidant and oxidative activity of NADES systems prepared from choline chloride, fructose, or glucose with a deep eutectic solvent and DES based on *N*,*N*-diethyl ethylammonium chloride and triethylene glycol, using dihydroethidium (DHE) as a probe to measure superoxide production in treated cells. It was observed that NADES exhibited lower redox stress compared to DES (Mbous et al. 2017).

Other assays attribute antioxidative properties or enhancement of the antioxidation process to these systems. One work mentions the use of a NADES formed by propanediol/ChCl/ water (1:1:1) as a solubilization medium for some antioxidants such as decyl rosmarinate or sinapine. The results obtained reflect how the NADES formulation improved the antioxidant activity compared to the ROS inhibition capacity of antioxidants dissolved in organic solvents (Durand et al. 2017). On the other hand, Martínez et al. studied the THEDES limonene:ibuprofen (1:4) and observed how the lowest tested concentration protected HT29 cells from oxidative stress by inhibiting the production of ROS and NO (Martínez et al. 2022).

A positive correlation corresponds to an increasing relationship between the assays. For these compounds, we observed a positive correlation among the enzymes SOD, CAT, and GPx, suggesting a coordinated response of the antioxidant system (Ighodaro and Akinloye 2018).

These enzymes are overexpressed compared to the control, indicating an antioxidant response to oxidative stress. In some cases, the significant increase in MDA (lipid peroxidation) may indicate that this attempt to counteract reactive oxygen species is not efficient revealing alteration in lipid membranes (Sánchez-Rodríguez et al. 2004).

This occurs at higher concentrations for the compounds **N00Cl-000** and **N00Cl-200** and at concentrations of 1000 mg/l for the compounds **N00Cl-400** and **N00Cl-3F00**.

In summary, these findings highlight the intricate interaction of DES with multicellular organisms and stress the importance of considering environmental factors and specific DES properties when assessing their toxicity and effects on biological systems.

## Conclusions

In this study, the effects of exposing *P. varians* to several DES were analyzed, revealing significant insights into the environmental impact of these substances. Notably, this research highlights the novel finding of a clear positive correlation between shrimp mortality and DES exposure concentration, with the results of LC50 displaying an odd–even effect. Among the DES tested, N00Cl-300 was identified as the least toxic, with LC50 values for all systems remaining above 1000 mg/L. The odd–even toxicity effect has been identified as a potential mechanism, suggesting that even-length chains might possess greater symmetry and pack more efficiently in cell membranes or biological binding sites compared to odd-length chains.

The study also provides new evidence of the potential mechanisms of toxicity, particularly related to oxidative stress. The imbalance observed between reactive oxygen species (ROS) and the antioxidant capacity of the organisms suggests that DES exposure may disrupt the cellular redox balance. Interestingly, for N00Cl-000, N00Cl-100, N00Cl-200, and N00Cl-300, biomarkers of oxidative stress were not altered at low concentrations, indicating that these DES can be considered safe up to 100 mg/l. However, at higher concentrations, significant toxicity was observed, likely due to oxidative stress, emphasizing the need for a precautionary approach in the use of these substances. While our study provides an important foundation for assessing the toxicity of DES, additional research is needed to fully understand their toxicological impact. This includes evaluating long-term effects, exploring a broader range of DES, and examining potential synergistic interactions to better assess environmental risks and develop effective mitigation strategies.

## Supplementary Information

Below is the link to the electronic supplementary material.

Supplementary file1 (DOCX 452 KB)

## Data Availability

Data will made available on request.
