# Supplementary material for: Assessment of oxidative stress biomarkers in Palaemon varians exposed to deep eutectic systems
Source: Environ Sci Pollut Res Int. 2024 Sep 21;31(47):57959–72. doi: 10.1007/s11356-024-34983-3 (PMC11467075; doi:10.1007/s11356-024-34983-3)
Supplement: Supplementary file 1 — Supplementary file1 (DOCX 452 KB) [file 11356_2024_34983_MOESM1_ESM.docx]

**Supplementary material**

Figure S1. Normalized mortality rate of studied DES at different concentrations: 0 mg/L (), 100 mg/L (),500 mg/L (), 1000 mg/L (), 2500 mg/L () and 5000 mg/L ().


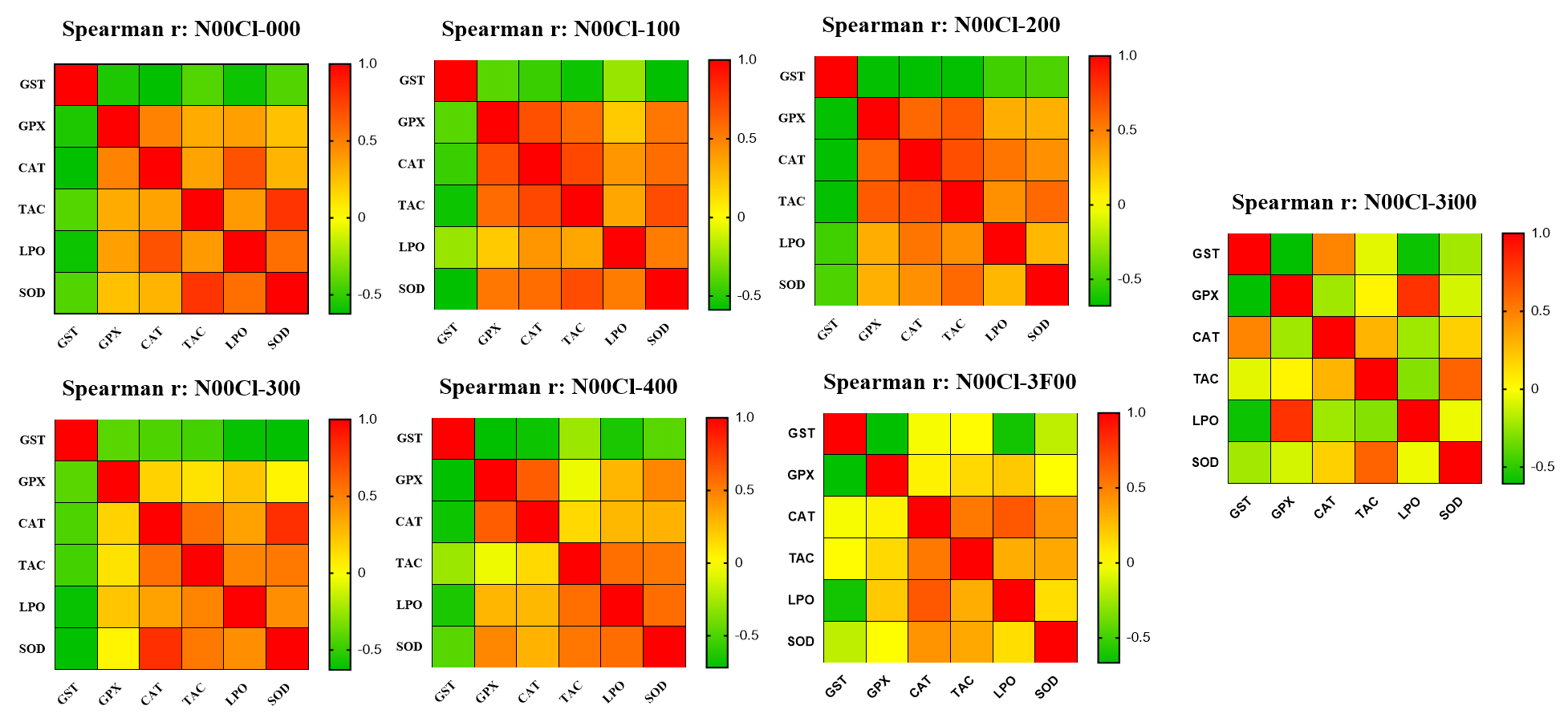


Figure S2. Correlation between enzymatic essays for each DES (Spearman correlation).
